# Relationship of Lifestyle Medical Advice and Non-HDL Cholesterol Control of a Nationally Representative US Sample with Hypercholesterolemia by Race/Ethnicity

**DOI:** 10.1155/2012/916816

**Published:** 2012-10-15

**Authors:** Joan Anne Vaccaro, Fatma G. Huffman

**Affiliations:** ^1^Department of Dietetics and Nutrition, MMC AHC 1-450, Florida International University, Miami, FL 33199, USA; ^2^Department of Dietetics and Nutrition, MMC AHC 1-435, Florida International University, Miami, FL 33199, USA

## Abstract

*Objective*. The main purpose of this study was to evaluate the associations of lifestyle medical advice and non-HDL cholesterol control of a nationally representative US sample of adults with hypercholesterolemia by race/ethnicity. *Methods*. Data were collected by appending sociodemographic, anthropometric, and laboratory data from two cycles of the National Health and Nutrition Survey (2007-2008 and 2009-2010). This study acquired data from male and female adults aged ≥ 20 years (*N* = 11,577), classified as either Mexican American (MA), (*n* = 2173), other Hispanic (OH) (*n* = 1298), Black non-Hispanic (BNH) (*n* = 2349), or White non-Hispanic (WNH) (*n* = 5737). *Results*. Minorities were more likely to report having received dietary, weight management, and exercise recommendations by healthcare professionals than WNH, adjusting for confounders. Approximately 80% of those receiving medical advice followed the recommendation, regardless of race/ethnicity. Of those who received medical advice, reporting “currently controlling or losing weight” was associated with lower non-HDL cholesterol. BNH who reported “currently controlling or losing weight” had higher non-HDL cholesterol than WNH who reported following the advice. *Conclusion*. The results suggest that current methods of communicating lifestyle advice may not be adequate across race/ethnicity and that a change in perspective and delivery of medical recommendations for persons with hypercholesterolemia is needed.

## 1. Introduction

Cholesterol, the functional unit of numerous, essential hormones and steroids in the human body, circulates in blood. Even though cholesterol is necessary for body function, elevated levels can result in atherosclerosis and cardiovascular disease. Levels of serum cholesterol less than 200 mg/dL are considered in the healthy, normal range; borderline cholesterol is 200–239 mg/dL; elevated blood cholesterol, 240 mg/dL or above, is classified as high cholesterol [1]. Primary goals of therapy and treatment are focused on low-density lipoprotein cholesterol (LDL-C) < 100 mg/dL, where 130 mg/dL is considered borderline high [1]. Despite the decrease in LDL-C since the 1960s, hypercholesterolemia, a key risk factor of atherosclerosis and coronary heart disease, currently affects nearly half of the US adult population [2]. Hypercholesterolemia is a metabolic disorder characterized by high levels of serum cholesterol, particularly LDL-C. For treatment purposes, hypercholesterolemia individually diagnosed based on high LDL-C and concurrence of other cardiovascular disease risk factors such as smoking, hypertension, diabetes, and a family history of premature coronary heart disease [3]. The recommended treatment of hypercholesterolemia involves weight loss, dietary and physical activity changes, and a possible medical regime. Prescription of LDL-C lowering medications should be reserved for aggressive treatment when LDL-C levels do not respond to dietary modifications and increased exercise [3, 4]. The side effects of statins, a commonly prescribed cholesterol-lowing medication, may outweigh their benefit for otherwise healthy adults [4]. 

A diagnosis of hypercholesterolemia and consequent treatment is only possible for adults who had adequate medical care, which included blood collection for a lipid panel. Among a nationally representative sample of US adults in 2005-2006, aged 20 years or older, only three-quarters reported ever having their cholesterol checked with lower reports for Mexican Americans and non-Hispanic Blacks as compared to non-Hispanic Whites [2]. Minorities have been reported to have less access and quality of health care for cholesterol screening and treatment [5]. Even when treated for hypercholesterolemia, a lower percent of minorities were prescribed dietary and exercise counseling, based on a representative US sample of approximately 27 million adults, aged ≥20 years, from a medical registry [5].

Because the initial treatment of hypercholesterolemia involves dietary and physical activity changes, it is imperative that the diagnosed individuals recall having received these lifestyle medical recommendations. Therefore, the aim of this study was to examine a US representative sample of adults for the years 2007–2010 and to investigate the following (1) the likelihood of lifestyle medical advice received differing by race/ethnicity for those adults who had their cholesterol checked; (2) percents of adults given and following medical advice; (3) the association of non-HDL cholesterol with reporting following lifestyle medical advice.

## 2. Materials and Methods

### 2.1. Data Collection

All data used for this study were approved by the research ethics board and publically available from appended 2-year cycles of datasets from the National Health and Nutrition Examination Survey (NHANES) 2007-2008 and 2009-2010 [6]. NHANES uses a complex, multistage, probability sample design to obtain representative samples of the noninstitutionalized, civilian US population [6]. This study acquired data from male and female adults aged ≥20 years (*N* = 11, 577). The final sample size for participants with hypercholesterolemia and measurements of total and/or non-HDL cholesterol was 3325 (MA = 505; OH = 362; BNH = 614; WNH = 1844).  

### 2.2. Major Variables

*Hypercholesterolemia* was considered an affirmative answer to “ever told by a doctor or other health care professional that you had a high blood cholesterol level”. Non-HDL cholesterol was constructed by subtracting HDL-C from total cholesterol. *Cholesterol control* (yes/no) was constructed as a binary variable with non-HDL cholesterol <130 mg/dL considered adequate control versus ≥130 mg/dL as inadequate control. The number of participants with this cutoff was chosen based on the National Cholesterol Education Program's (NCEP-ATP III) guideline of 130 mg/dL for persons with >20% cardiovascular disease risk [1] and the American Association of Clinical Endocrinologists' (AACE) guidelines of non-HDL cholesterol as 30 mg/dL above the goal (<100 mg/dL) for LDL-C [7]. *Medical advice* (yes, no) for weight, diet, and exercise were the major independent variables and phrased as follows: to lower your blood cholesterol have you ever been told by your doctor or other health professional to: (1) eat fewer high-fat or high-cholesterol foods? (2) control weight or lose weight? (3) increase physical activity or exercise?

### 2.3. Adjustment Variables

Comorbidities of hypercholesterolemia used in the analysis were coronary heart disease, diabetes, and hypertension. Coronary heart disease was defined as an affirmative response to the question, “Has a doctor or other health professional ever told you that you had coronary heart disease?” Diabetes was considered an affirmative answer to the question, “Has a doctor or other health care professional told you that you had diabetes or sugar diabetes?” Having hypertension was defined as a systolic blood pressure ≥140 mm Hg, a diastolic blood pressure ≥90 mm Hg (using the average of first two readings), or taking hypertension medication [8].

Obesity was estimated using the proxy measurement of body mass index (BMI) and was calculated from measured weight and height (kg/m^2^) and was categorized based on the National Institutes of Health's recommended levels: normal weight (<24.9 kg/m^2^); overweight (24.9–29.9 kg/m^2^); obese I (30.0–34.4 kg/m^2^); obese II or more (≥35 kg/m^2^) [9]. A binary variable was created for current cigarette use versus not currently smoking. Education was assessed as < high school; high school diploma or general equivalency diploma; > high school. Health insurance was defined as having any coverage within the past 12 months (yes/no).

### 2.4. Statistical Analysis

Chi-square tests were performed to determine hypercholesterolemia and medical advice by ethnicity. Logistic regression analyses were conducted for non-HDL cholesterol control as the outcome variable. Reduced models included race/ethnicity adjusted for age and gender. Fully adjusted models included the covariates of the simple models along with: currently smoking, BMI, education, health insurance, and lipid-lowering medication. In addition, hypertension, diabetes, and coronary heart disease were added to test medical advice received by race/ethnicity. Adjusted sample weights were used to account for unequal probabilities of selection and nonresponse in the multistage stratified cluster sampling design used in NHANES in order to achieve unbiased national estimates. The sample weights used were based on the data file with the smallest sample size, the Mobile Examination Center (MEC), and were computed using the average of the 2-year sample weights for each cycle, as per guidelines set by NHANES [10]. Data analysis was conducted with complex module, IBM-SPSS version 19. The Wald *F* statistic was used to determine model significance for logistic regression analysis [11]. For the chi-squared test, significance was based on the adjusted *F* statistic, a variant of the second-order Rao Scott adjusted chi-square statistic used for analyses with sample weights, where df1 + df2 = 33 (total degrees of freedom).

## 3. Results

Key risk factors associated with hypercholesterolemia are shown in Table 1. There were significant differences in major sociodemographics, body mass index, morbidities, and having cholesterol checks among race/ethnicity. WNH had the highest prevalence of reporting having their cholesterol checked followed by BNH, OH, and MA. For those with hypercholesterolemia, coronary heart disease was higher in WNH than other race/ethnicity categories. BNH, followed by WNH, had higher prevalence of hypertension as compared to Hispanics. BNH had a higher prevalence of obesity II [BMI ≥ 35(kg/m^2^)] and diabetes than other groups. A lower percent of minorities were covered by health insurance with MA having the least coverage.

The likelihood of receiving medical advice by race/ethnicity is shown in Table 2. Compared to WNH, MA and BNH were more likely to be given advice to eat fewer high-fat or –cholesterol foods. MA and OH were more likely to be told to lose or control their weight as compared to WNH. All Hispanics and BNH were more likely to be told to increase physical activity or exercise as compared to WNH.

The percent of adults with hypercholesterolemia who reported affirmatively that they were given diet, weight, and exercise medical advice to reduce their cholesterol levels and who reported affirmatively now following the advice is shown in Table 3. Despite the fact that weight management and increasing physical activity are vital recommendations for individuals with hypercholesterolemia, approximately 40% and 30%, respectively, reported not receiving advice for these lifestyle behaviors. The majority of the participants (approximately 80%), who reported having received medical advice, followed the corresponding recommendations. 

Participants who responded affirmatively to being given medical advice for hypercholesterolemia were included in a chi-squared test of whether they followed each recommendation (yes/no) across non-HDL-C control (<130 mg/dL, yes/≥130 mg/dL, no) (Table 3). Reporting now eating fewer fatty foods (*P* = .002) and now losing weight (*P* = .005) were significantly associated with the corresponding recommendation. Cholesterol control (non-HDL-C < 130 mg/dL) was found for approximately two-thirds of those who reported eating fewer fatty foods (67.5%, SE = 1.2%). Similarly, of those participants who reported now controlling or losing weight, 67.3% (SE = 1.4%) had cholesterol control (non-HDL-C < 130 mg/dL).

Reporting following medical recommendations for diet and physical activity (yes/no) and non-HDL cholesterol (continuous) was tested by a complex general linear model. The model was significant; however, of the behaviors only “now controlling or losing weight” was significant (data not shown). Reduced and full models were constructed for reporting “controlling or losing weight” by race/ethnicity (Table 4). Lower non-HDL cholesterol levels were associated with an affirmative report of “now controlling or losing weight” for the reduced model (adjusted only for age and gender (*P* = .001)). For the fully adjusted model, which included, BMI, health insurance, education, currently smoking, and lipid-lowering medication(s), the 2-way interaction “controlling or losing weight” by race/ethnicity was significant (*P* = .014) and the main effect (controlling or losing weight) was no longer significant (*P* = .998); however race/ethnicity remained significant (*P* < .001). BNH who reported “controlling or losing weight” had significantly higher non-HDL cholesterol as compared to WNH (*P* = .003).

Use of lipid-lowering medications did not negate the association of losing weight with non-HDL cholesterol as a continuous variable; however, for logistic regression models lipid-lowering medications did negate the likelihood of having cholesterol control (non-HDL cholesterol < 130 mg/dL); albeit the results were marginal [OR = 1.49 (1.18, 1.76), with independents: race, gender, age, BMI, health insurance, education, and smoking; OR = 1.18 (0.93, 1.49) with the same independents and the additional covariate, lipid-lowering medications] (data not shown).

## 4. Discussion

In a nationally representative population of adults with hypercholesterolemia, we found discrepancies in medical advice for diet, weight management, and exercise by race/ethnicity. Reporting having received medical advice was associated with performing the recommended behavior; yet, over one-third of adults across race/ethnicity with hypercholesterolemia reported not receiving dietary, weight management, or exercise recommendations by either their physician or other health care professional. Moreover, there were differences by race/ethnicity in reporting having received these lifestyle recommendations. These results suggest confounders such as patient-provider relationships and health beliefs, which may vary by race/ethnicity and may also influence interpretation of advice and resulting health outcomes. 

African Americans, as compared to WNH with comparable healthcare coverage, were found to have a poorer quality of patient-provider relationships as well as poorer control of cholesterol, in a national, multicentered prospective study of patients with diabetes [12]. In the current study, BNH who reported “controlling or losing weight” had significantly higher non-HDL cholesterol as compared to WNH. This disparity may be the result of differences in perceived vulnerability or susceptibility by race/ethnicity. In this case, BNH may not perceive danger of cardiac events until their cholesterol level in a higher range than their WNH counterparts.

The Agency for Health Care Research and Quality (AHRQ) [13] calculated 70% disparity (worsening or no improvement) between minorities and WNH with respect to quality of care change in five years (from 2003 to 2008). In contrast to the findings of Willson et al. [5], minorities were more likely to receive medical advice than WNH in our analysis; however, patient-provider relationships were not assessed and may have confounded the results. It has been suggested that physicians who positively engage minority patients may either be members of a minority group or have had experiences dealing with minority patient's health barriers and may be acting in an attempt to reduce health disparities [14]. Piette et al. [15] found that African Americans and Spanish-speaking patients as well as patients with a lower education reported having better communication with their physicians than patients of other race/ethnicities. Conversely, African American patients reported poorer patient-physician communication as compared to Whites with the same healthcare coverage in a national multicenter prospective study [12]. The investigators found that health outcomes and disease control had been positively associated with the quality of patient-physician communication, as measured by the patients' trust and report of physicians listening, explaining, showing respect, and spending time [12]. 

In the current study, there were no differences in LDL-C managing behaviors among race/ethnicity for adults with hypercholesterolemia given lifestyle medical advice. Instead, being given medical recommendations was associated with making the changes. These findings support the benefit of secondary prevention measures of diet and exercise recommendations by medical professionals. Several studies found racial/ethnic differences in therapeutic lifestyle changes for patients with chronic disease. BeLue et al. [16] reported different patterns of diet-exercise behavior (fruit and vegetable intake, fat intake and exercise) by race/gender groups for Black and White adults with hypercholesterolemia. Oster et al. [17] reported that Blacks and Hispanics were less likely to monitor their diet than WNH with diabetes in a national managed care organization. Moreover, for patients with diabetes, Blacks were less likely to exercise as compared to Whites [17, 18].

Weight loss, but not reducing fatty foods nor increasing physical activity, was associated with lower non-HDL cholesterol in our study. Several meta-analyses of dietary interventions for cardiovascular risk reduction have reported modest effects in cholesterol reduction [19–21]. Albeit, even modest reductions in serum cholesterol, about 10%, have been associated with a 50% decrease in the incidence of coronary heart disease for age 40 and 20% for age 70 for approximately 500,000 men in a meta-analysis of cohort studies [22]. 

In order for serum non-HDL cholesterol management to be effective, several precursors are necessary: (1) access to quality medical care that provides a diagnosis of hypercholesterolemia; (2) a culturally appropriate, nondiscriminatory environment that encourages continuation of healthcare utilization; and, (3) effective patient-provider relationships that involve patients in their treatment plans and empower them to follow disease-specific recommendations. Barriers for receiving and interpreting medical advice may differ by race/ethnicity. Based on a national survey of healthcare quality, African Americans, Hispanics, and Asians reported more perceived healthcare provider discrimination than WNH [23]. Poorer health status was associated with perceived healthcare discrimination more frequently in minorities than WNH [23]. There is inconclusive evidence regarding differences in patient-provider communication among the Hispanic subgroups. Although access to healthcare varies among Hispanic groups [24], the differences in access were not related to the quality of communication between the patient and their healthcare provider [25].

Receiving advice may not be interpreted equally across patients and may be a confounder in serum non-HDL cholesterol control. In particular, the interpretation of the medical recommendation to “eat fewer high-fat or high-cholesterol foods” may not be consistent among patients and may vary by race/ethnicity. Since people usually eat *ad libitum* (until they are satisfied), they are likely to exchange one food for another, rather than to reduce total calories [26]. In an effort to reduce “fatty foods,” patients may substitute any combination of the following: refined carbohydrates (such as white flour or white rice), unrefined grains (such as whole oats, wheat, or brown rice), or healthy fats (such as nuts, seeds, avocados, and mono- and poly-unsaturated oils) for “fatty foods.” The range of actions resulting in the interpretation of this dietary advice may have been a confounder in non-HDL cholesterol control. 

Numerous prospective studies of both primary and secondary preventive measures to reduce the prevalence of hypercholesterolemia suggest that these efforts may prove to be beneficial by reducing its prevalence in the US. Healthy adults followed for 27 years were 26% and 30% less likely to develop hypercholesterolemia that maintained or increased their fitness level, respectively, compared to those who lost fitness levels [27]. Secondary prevention, aimed at non-HDL cholesterol control for those with hypercholesterolemia, also entails medical recommendations for diet, weight, and physical activity; yet, research regarding the efficacy of advice with behavior changes and health outcomes for persons with hypercholesterolemia is limited.

Our results should be interpreted with respect to the study strengths and limitations. A major strength of this study was the use of a large, nationally representative dataset. Another strength of this study was the use of non-HDL cholesterol as both a binary and continuous variable to measure cholesterol control as opposed to total cholesterol. The use of non-HDL cholesterol <130 mg/dL as adequate control may not be a suitable goal for all persons with hypercholesterolemia. The AACE guidelines are that goals for non-HDL cholesterol be set at 30 mg/dL above the target for LDL-C [7]. Although a target of non-HDL cholesterol <130 mg/dL is recommended for most patients, guidelines for lipid goals for patients at risk for coronary artery disease vary by physician and should be set as 30 mg/dL above the patient's LDL-C goals [7]. Several limitations were noted. The category “Others” was not used due to a small sample size. Data concerning having serum cholesterol checked and being given medical advice to manage hypercholesterolemia were self-reported and may contain some inaccuracies. Moreover, bias by race/ethnicity, age, gender, or other characteristics are possible due to recall differences among people. 

## 5. Conclusions

The findings of this study indicate a lack of congruence in following medical advice and achieving adequate non-HDL cholesterol control by race/ethnicity. These results suggest that current methods of communicating lifestyle advice may not be adequate across ethnicity/race and that a change in perspective and delivery of medical recommendations for persons with hypercholesterolemia is needed. Evaluation of patient-provider communication with cholesterol management across race/ethnicity may be necessary to develop effective dietary and physical activity intervention strategies. The results of our study may inform healthcare professionals of the need to develop client-centered treatment plans with clear, individualized goals for diet, weight management, and physical activity.

## Figures and Tables

**Table 1:** Major hypercholesterolemia risk factors of the study population by race/ethnicity.

Variable	MA	OH	BNH	WNH	*P* value
Cholesterol checked (yes)	51.2 (1.6)^a^	60.5 (2.2)^b^	69.7 (1.3)^c^	78.5 (0.9)^d^	<.001

Inadequate non-HDL-C (≥130 mg/dL)	25.8 (2.0)^a^	22.1 (2.5)^a^	33.1 (2.1)^b^	30.6 (1.1)^b^	.008
High cholesterol (≥200 mg/dL)	45.9 (1.4)^a^	42.1 (2.2)^a,b^	39.7 (1.3)^b^	44.5 (0.9)^a,b^	.019
Lipid-lowering medications (yes)	50.7 (3.4)^−^	44.1 (3.4)^−^	51.7 (1.8)^−^	54.7 (1.6)^−^	.129
Age (years)	51.2 (1.2)^a^	52.3 (1.1)^a,b^	53.8 (0.5)^b^	57.4 (0.3)^c^	<.001
Education: <HS	51.5 (1.6)^a^	37.7 (2.4)^b^	26.1 (1.7)^c^	13.8 (1.4)^d^	<.001
No health insurance	52.2 (1.9)^a^	36.3 (3.1)^b^	26.0 (1.4)^c^	13.7 (0.7)^d^	<.001
Current smoker	11.4 (1.1)^a^	14.5 (1.8)^a^	21.7 (1.7)^b^	19.1 (1.2)^b^	<.001
BMI 30–34.9 (kg/m^2^) obesity I	22.5 (1.6)^a^	18.6 (1.4)^b,c^	21.6 (1.3)^a,c^	18.5 (0.6)^b^	<.001
BMI ≥ 35(kg/m^2^) obesity II	18.0 (0.8)^a^	17.0 (1.3)^a^	26.1 (1.2)^b^	16.2 (0.6)^a^	<.001
HTN (yes)	19.2 (1.4)^a^	21.1 (1.6)^a^	38.7 (1.3)^b^	31.8 (1.0)^c^	<.001
Diabetes (yes)	8.4 (0.8)^a^	8.5 (0.8)^a^	13.5 (0.8)^b^	7.6 (0.6)^c^	<.001
CHD (yes)	1.8 (0.3)^a^	1.4 (0.3)^a^	1.6 (0.2)^a^	3.7 (0.2)^b^	<.001

MA: Mexican American; OH: other Hispanic, not Mexican; BNH: Black non-Hispanic; WNH: White non-Hispanic; non-HDL-C: non-HDL cholesterol (total cholesterol minus HDL-C); BMI: body mass index; HTN: hypertension; CHD: coronary heart disease.

The data represent the weighted percent and standard error [% (SE)]. Significance is based on the adjusted *F*, a variant of the second-order Rao-Scott adjusted chi-square statistic and its degrees of freedom. Each superscript letter denotes a subset of race/ethnicity categories whose column proportions do not differ significantly from each other at the 0.05 level for each row (variable). Significant differences between each group were measured by complex logistic regression analyses across race/ethnicity separately for each dependent binary variable. Age was tested by complex general linear models, recoding ethnicity to measure differences between groups.

**Table 2:** Odds ratios for receiving medical advice by race/ethnicity.

Dependent variable	Independent	Model 1	Model 2	Model 3
Told to eat less fat	MA	1.48 (1.09, 2.04)	1.59 (1.14, 2.20)	1.60 (1.14, 2.26)
	OH	1.10 (0.81, 1.48)	1.13 (0.82, 1.56)	1.15 (0.82, 1.60)
	BNH	1.70 (1.36, 2.12)	1.59 (1.37, 1.99)	1.49 (1.19, 1.86)
	WNH	1.00	1.00	1.00

Told to control or lose weight	MA	1.54 (1.12, 2.12)	1.58 (1.12, 2.21)	1.55 (1.07, 2.25)
	OH	1.75 (1.33, 2.31)	1.77 (1.32, 2.39)	1.75 (1.26, 2.41)
	BNH	1.37 (1.11, 1.70)	1.38 (1.11, 1.71)	1.21 (0.95, 1.54)
	WNH	1.00	1.00	1.00

Told to exercise	MA	1.65 (2.23, 2.22)	1.64 (1.21, 2.23)	1.64 (1.20, 2.26)
	OH	1.56 (1.20, 2.04)	1.56 (1.19, 2.06)	1.54 (1.15, 2.07)
	BNH	1.71 (1.41, 2.08)	1.51 (1.24, 1.85)	1.35 (1.09, 1.68)
	WNH	1.00	1.00	1.00

Model 1: adjusted for age and gender (and BMI for “told to control or lose weight”).

Model 2: adjusted for age, gender, BMI, current smoker, education, and health insurance.

Model 3: adjusted for age, gender, BMI, current smoker, education, health insurance, coronary heart disease, diabetes, and hypertension.

**Table 3:** Percent of adults given and following medical advice.

Given the recommendation to lower serum cholesterol	Percent given the advice who are following the recommendation	SE
Told to eat less high-fat or high-cholesterol foods 81.8% (yes)	82.8 (80.7, 84.7)	1.0
Told to control weight or lose weight 59.4% (yes)	84.0 (81.3, 86.3)	1.2
Told to increase physical activity or exercise 69.9% (yes)	78.0 (75.4, 80.3)	1.2

Results are given as [mean (95% CI) SE].

**Table 4:** Effect of reporting “now controlling or losing weight” on non-HDL cholesterol of those participants who were told to “control or reduce weight” by race/ethnicity.

Independent variables	Model 1		Model 2	
	B (95% CI)	SE	B (95% CI)	SE
Now controlling or losing weight (yes)	−8.9 (−13, −4.5)	2.2*	−4.4 (−8.4, −1.3)	1.8
MA	2.2 (−5.8, 10.2)	3.9	−1.1 (−9.1, 6.8)	3.9
OH	−2.9 (−12, 6.5)	4.7	−3.8 (−12, 4.6)	4.1
BNH	−16 (−22, −11)	2.8*	−18 (24, 11)	3.2*
Lose wt by MA	0.70 (−9.9, 11)	5.2	0.66 (−8.2, 9,5)	4.3
Lose wt by OH	9.7 (−0.32, 19)	4.9	5.0 (−4.0, 14)	4.4
Lose wt by BNH	15 (6.8, 23)	4.1*	14 (5.2, 22)	4.2*

Model summary	*R* ^2^ = 0.063		*R* ^2^ = 0.21	
	*F*(9, 24) = 41.0, *P* < 0.001		*F*(17, 16) =58.7, *P* < 0.001	

Non-HDL cholesterol: non-high-density lipoprotein cholesterol; MA: Mexican American; OH: other Hispanic; BNH: Black, non-Hispanic; wt: weight.

Model 1 was adjusted for gender and age. Model 2 included body mass index, health insurance, education, currently smoking, and taking cholesterol-lowering medication(s) along with the adjustments of Model 1. White non-Hispanic (WNH) is the reference group for race/ethnicity. Significance (*P* < 0.05) is denoted by (∗).
